# Angiogenesis in Spontaneous Tumors and Implications for Comparative Tumor Biology

**DOI:** 10.1155/2014/919570

**Published:** 2014-01-19

**Authors:** C. Benazzi, A. Al-Dissi, C. H. Chau, W. D. Figg, G. Sarli, J. T. de Oliveira, F. Gärtner

**Affiliations:** ^1^Department of Veterinary Medical Sciences, University of Bologna, Via Tolara di Sopra 50, 40064 Ozzano Emilia, Bologna, Italy; ^2^Department of Veterinary Pathology, Western College of Veterinary Medicine, University of Saskatchewan, 52 Campus Drive, Saskatoon, SK, Canada S7N 5B4; ^3^National Cancer Institute, Bethesda, MD 20892, USA; ^4^Institute of Pathology and Molecular Immunology of the University of Porto (IPATIMUP), 4200-456 Porto, Portugal; ^5^Abel Salazar Institute of Biomedical Science, University of Porto (ICBAS-UP), 4200-456 Porto, Portugal

## Abstract

Blood supply is essential for development and growth of tumors and angiogenesis is the fundamental process of new blood vessel formation from preexisting ones. Angiogenesis is a prognostic indicator for a variety of tumors, and it coincides with increased shedding of neoplastic cells into the circulation and metastasis. Several molecules such as cell surface receptors, growth factors, and enzymes are involved in this process. While antiangiogenic therapy for cancer has been proposed over 20 years ago, it has garnered much controversy in recent years within the scientific community. The complex relationships between the angiogenic signaling cascade and antiangiogenic substances have indicated the angiogenic pathway as a valid target for anticancer drug development and VEGF has become the primary antiangiogenic drug target. This review discusses the basic and clinical perspectives of angiogenesis highlighting the importance of comparative biology in understanding tumor angiogenesis and the integration of these model systems for future drug development.

## 1. Introduction 

Blood is essential for tumor growth and progression and new vascular segments are needed to supply the growing tumor mass with oxygen and nutrients. Different forms of neovascularization are known, and the most important are vasculogenesis (defined as *de novo* formation of a capillary plexus by endothelial progenitor cells) [1]; angiogenesis (formation of a new capillary network from preexisting capillaries) [2]; vasculogenic mimicry (a special passage of blood without endothelial cells) [3]; and vessel cooption (a process where tumor cells initially coopt host blood vasculature without inducing angiogenesis; the coopted host vasculature regresses, leading to a secondary avascular tumor, hypoxia, and robust angiogenesis at the tumor margin) [4]. Tumors can use all the different modes of vessel formation and these different mechanisms may exist concomitantly in the same tumor or may be selectively involved in a specific tumor type or host environment [5].

It has been established that *vasculogenesis* occurs during embryogenesis, when endothelial cells are born from progenitor cell types [6], and also in the adult and particularly during tumor vascularization [7]. Vasculogenesis in tumors is *de novo *vessel formation by *in situ *incorporation, differentiation, migration, and/or proliferation of bone marrow-derived endothelial precursor cells [8, 9]. A wealth of evidence indicates that tumor blood vessels differ significantly from normal vessels in their structural organization and endothelial properties. This finding suggests that tumor vascularization depends on mechanisms distinct from the simple recruitment from adjacent tissue of preexisting blood vessels. The recapitulation of an embryonic-like vasculogenesis may allow a *de novo *formation of vessels within the tumor. This process involves endothelial differentiation of normal or malignant adult cells bearing stem and progenitor properties leading to generation of endothelial cells with abnormal characteristics. Tumor endothelial cells (TECs) may originate from tissue resident or bone marrow derived stem cells undergoing endothelial differentiation, or from tumor stem cells that can produce both tumor cells and TECs. These mechanisms suggest a contribution of vasculogenesis to neoformed blood vessels in tumors [7].

*Vasculogenic mimicry* (VM) was first described in aggressive melanoma by Maniotis et al. [3], who stated that the generation of patterned melanoma microcirculation is mediated by the tumor cells themselves and may function independently of tumor angiogenic mechanisms during various phases of tumor progression. The name was coined to describe the formation of these channels by aggressive tumor cells: vasculogenic, because the channels are not formed from preexisting vessels, and mimicry, because the channels are not true blood vessels, but merely mimic the function of vessels [10, 11]. In fact, it consists in *de novo* generation of microvascular channels by genetically deregulated, aggressive tumor cells without endothelial cell participation [10]. As shown by transmission electron microscopy, in melanoma the “vascular channel” is lined by a thin basal lamina corresponding to the wall of the vessel, but no endothelial cells are detected. Most of these channels seem to be connected to normal blood vessels [5].

In *vessel cooption*, known also as *mosaic vessel formation*, in contrast with the prevailing view that most tumors and metastases begin as avascular masses, evidence is presented that a subset of tumors initially grows by coopting existing host vessels. Vessels are surrounded, coopted by tumor cells, and no sprouts are observed [5]. This coopted host vasculature does not immediately undergo angiogenesis to support the tumor but instead regresses, leading to a secondarily avascular tumor and massive tumor cell loss. However, the remaining tumor is rescued by robust angiogenesis at the tumor margin [12]. The role of vessel cooption in tumorigenesis is still debated. Vajkoczy et al. [13] in the results of their study report the fact that the cellular organization of the solid tumor component did not follow the organization of the host blood vessels which clearly indicates that the majority of tumor cells do not coopt host blood vessels. According to the same authors, at this multicellular stage, the tumor cells have already initiated their neovascularization by inducing angiogenic sprouting from the microvasculature of the adjacent host tissue.

*Angiogenesis* is the most studied form of neovascular growth in cancer. As early as 1971, Judah Folkman proposed the hypothesis that tumor growth is dependent on the formation of new blood vessels. Angiogenesis is essential for the development and evolution of neoplastic disease, as both tumor growth and metastasis require persistent new blood vessels and ongoing angiogenesis is essential for rapid expansion of a tumor mass [6, 14]. Angiogenesis can be assessed as intratumoral microvessel density (IMVD), which is related to tumor aggressiveness, metastasis, and decreased patient survival [14]; therefore, inhibition of tumor angiogenesis would be an effective strategy to treat cancer [15]. In angiogenesis, new capillaries originate from existing vessels [16]. Induction of angiogenesis is a discrete component of the tumor phenotype, one that is often activated during the early, preneoplastic stages in the development of a tumor [6]. In the majority of cancers, vessel growth is not only stimulated, but these vessels are also abnormal in almost all aspects of their structure and function. Abnormal tumor vessels can also impede the function of immune cells in tumors, as well as the transport and/or distribution of chemotherapeutics and oxygen. Interstitial hypertension, hypoxia, and acidosis—which are all results of abnormal vessel structure and function—create a favorable environment for tumor progression and metastasis [8].

## 2. Different Mechanisms of Angiogenesis

It was originally considered that new blood vessel formation in tumors only occurred after such a tumor became invasive. However, it has been shown that angiogenic growth factors are already present in preinvasive lesions [17]. Epidemiological studies showed that patients bearing premalignant lesions have a high risk to develop an invasive cancer, and premalignant lesions can be found in almost all epithelial organs. These lesions are characterized by disordered proliferation, loss of cellular uniformity and architecture; some seem to be reversible, but others are progressive, and predictive factors of reversibility or progression are virtually unknown [18]. Angiogenesis precedes overt tumor formation during chemically induced carcinogenesis, suggesting that tumor progression depends on a switch from a prevascular to a vascular phase [6]. According to Folkman et al. [19], angiogenic activity first appears in areas of hyperplasia before the onset of tumor formation. Not all tumors are angiogenic from the beginning of their natural evolution and the angiogenic switch reflects the ability of the tumor and inflammatory cells to secrete angiogenic factors in tumor microenvironment [18]. Without the process of neovascularization, tumors remain in their dormant, nonangiogenic form, where proliferation is balanced with apoptosis, maintaining these microtumors quiescent [20]. The activation of the angiogenic switch takes place during the early stages of tumor development, suggesting that the regulation of angiogenesis is a discrete, potentially self-limiting step in the pathway to many solid tumors [6].

Marked differences are suggested among different types of solid tumors and among species in the promotion of a switch of the angiogenic type, depending on the action of proangiogenic factors or antiangiogenic inhibitors on endothelial or stromal cells that subsequently form capillary “sprouts” [4, 21, 22].

Sprouting angiogenesis is the first of two processes characterizing angiogenesis [23], the second being intussusceptive angiogenesis [24]. Sprouting angiogenesis is a process through which a single endothelial cell, called the tip cell, is selected from the vasculature, overcoming its quiescent environment and forming a new vessel [25]. Activation of sprouting is a relatively sluggish process *in vivo*, requiring more than 24 h, and at least 3–5 days elapse before a new capillary loop becomes perfused and is integrated into the vascular system [23, 26]. 

**Figure 1:**
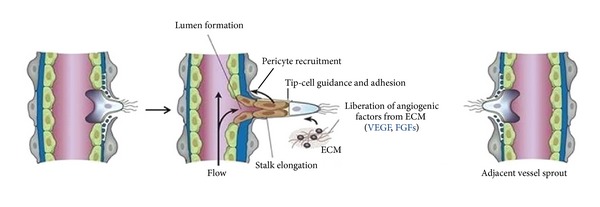
Angiogenetic sprouting: after stimulation with angiogenic factors, the quiescent vessel dilates and an endothelial cell tip cell is selected. Stalk cells behind the tip cell proliferate, elongate, and form a lumen, and sprouts fuse with an adjacent vessel sprout to establish a perfused neovessel, (from [8], modified).

The sprouting of new vessel segments, both in normal organ development and in tumors, follows a well-defined program that starts with degradation of basement membrane on the side of the tumoral postcapillary venule situated close to the angiogenic stimulus [23]; then the tip endothelial cell migrates towards a chemoattractant angiogenic signal constituted of growth factors that are secreted by the tumour cells and their stroma [25] and proliferates forming solid sprouts of endothelial cells connecting a neighboring vessel and restructuring of the sprout into a lumen lined by endothelial cells and integrated in the vascular network (Figure 1) [23]. Because sprouting angiogenesis is an invasive process, proteolytic activities are required. Proteolysis is largely mediated by matrix metalloproteinases (MMPs), a family of zinc containing calcium-dependent endopeptidases secreted by various cell types [27]. The role of the metalloproteinase MT1-MMP (membrane-type 1 matrix metalloproteinase) was addressed in different cell types in terms of its ability to regulate sprout formation [25]. Enzymes are needed not only for the degradation of the basement membrane of endothelial cells allowing invasion into the tissue, but also for cell migration and removal of obstructing matrix proteins and for creating space in the matrix to allow generation of endothelial cell tubules [28, 29]. MT1-MMP, other MMPs, and the related ADAMs (a disintegrin and metalloproteinase domain) modulate the balance between pro- and antiangiogenic factors by activation and modification of growth factors and chemokines, ectodomain shedding with accompanied receptor activation, shedding of cytokines from membrane-bound precursors, and generation of (matrix) protein fragments that inhibit or activate angiogenesis [29].

After migrating, endothelial cells proliferate during the sprouting process in tumors; they maintain their basal-luminal polarity and form a slit-like lumen that is continuous with the lumen of the so-called mother vessel. Basement membrane material is deposited continuously by the sprout endothelial cells, whereas only the tip of the growing bud is in contact with the collagenous connective tissue matrix [30]. As the final step, proliferating pericytes of the mother vessel migrate along the basement membrane of the sprout, resulting in the maturation of the new vessel [30].

In contrast to endothelial sprouting, the other major angiogenic mechanism is intussusceptive microvascular growth, or intussusceptive angiogenesis, which is achieved by intraluminal growth. Intussusceptive angiogenesis has been described in a wide variety of normal and pathological conditions, is faster, and does not depend primarily on endothelial cell proliferation. It occurs within hours or even minutes, does not primarily need endothelial cell proliferation, and can expand to all existing capillary networks [1, 24]. Djonov et al. (2001) demonstrated that in mammary tumors of neuT transgenic mice, both sprouting and intussusceptive angiogenesis occur simultaneously in the same nodule. The transient normalization of tumor vasculature may be explained on morphogenic level by the angiogenic switch from sprouting to intussusception. The switch to intussusceptive mode of angiogenesis improves the perfusion of the tumor mass as has been shown by the decrease in hypoxia of the tumor mass [31].

The concept of network expansion and vessel formation was introduced by Caduff et al. [32] when they studied the developing microvasculature in the postnatal rat lung. Obviously, this concept represented a new mode of angiogenesis—an alternative to capillary sprouting. The capillary system would expand “within itself.” The most appropriate term for such a mechanism in histology was intussusception, and thus this concept was named intussusceptional microvascular growth (IMG) [32]. This terminology was modified to intussusceptive angiogenesis by Burri and Tarek [33], who demonstrated the tissue posts to be pillars in the vascular lumina of developing vessels by serial sectioning [24] (known also as “nonsprouting or splitting angiogenesis”) [34].

**Figure 2:**
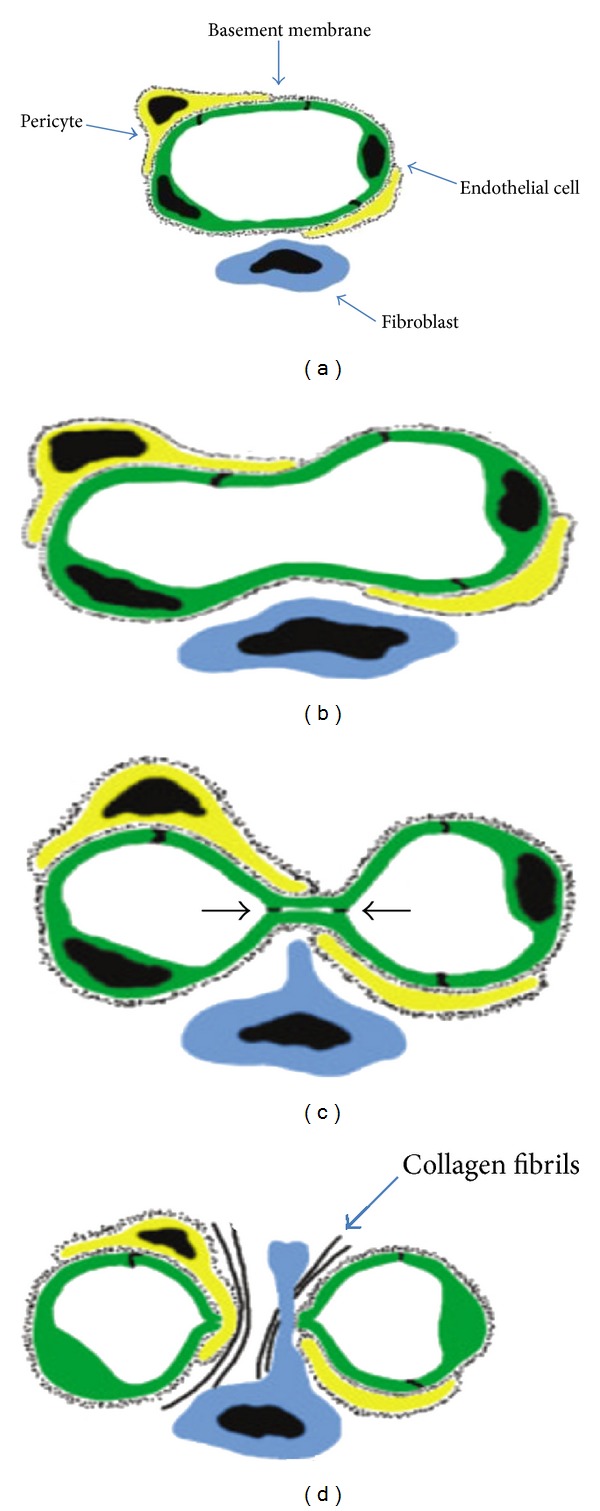
Intussusceptive angiogenesis. Two opposite endothelial cell membranes make contact (“kissing” contact) and interendothelial junctions develop at their edge. Connective tissue columns, called tissue pillars, grow into the lumen between the endothelial contact, resulting in partitioning of the vessel lumen (from [28]; modified).

The most characteristic feature of intussusceptive angiogenesis in tumor tissues is thought to be the development of protrusions or infoldings of the vessel wall within the lumen [1] (Figure 2). According to Auguste et al. [5], two opposite endothelial cell membranes make contact (“kissing” contact) and interendothelial junctions develop at their edge. In the middle of the kissing contact, membranes are thinned and pressure induced by the cytoplasm opens them and separates the two vessels.

The next step is the insertion of connective tissue columns, called tissue pillars, into the lumen between the endothelial contact and the subsequent growth of these pillars, resulting in partitioning of the vessel lumen and the consequent increase in the density of the given capillary network [5, 30]. Pillars, the hallmark of intussusceptive angiogenesis, develop within capillaries, small arteries, and veins and subsequently fuse, thus delineating new vascular entities or resulting in vessel remodeling [24]. Intussusceptive angiogenesis therefore splits an existing lumen into two [35]. The mechanism of connective tissue pillar formation during tumor-induced intussusceptive angiogenesis can be summarized as follows. First, transluminal endothelial bridges are formed. Second, collagen bundles adjacent to the vessel are seized by the abluminal side of a bridge forming endothelium. Finally, maturation of these nascent pillars occurs via the migration of pericytes and myofibroblasts into the collagen core of the pillar and the deposition of additional collagenous connective tissue by these cells. Tumor cells are able to both incorporate into the pillars and contribute to their growth; therefore, they help to dilute the newly formed capillary network [30].

Intussusceptive angiogenesis may have different outcomes depending on the location of the pillars, and different subtypes are named as follows: intussusceptive microvascular growth (IMG), which refers to the expansion of the capillary bed, and intussusceptive arborization (IAR) that describes the formation of the typical vascular tree, while intussusceptive branching remodeling (IBR) denotes vascular remodeling and adaptation to suit local perfusion requirements and includes intussusceptive vascular pruning (IVP). Vascular pruning is an essential adaptive mechanism resulting in the regression of excessive vascular branches and the creation of hierarchical, thermodynamically efficient angioarchitecture [36]. It can be summarized as the formation of multiple eccentric pillars at the bifurcation points and their subsequent successive fusions, which leads to partial and later to total luminal obstruction and separation (cutting-off) of one or more of the affected daughter branches. At the initial stage, intraluminal protrusions of endothelial cells lying at the opposing sides of the vessel are formed. The contact between them has to be established for the single pillar to emerge [36].

In the majority of cancers, vessel growth is not only stimulated, but the new vessels are also abnormal for structure and function. These vessels are tortuous, irregularly shaped, and hyperpermeable [37]. Tumor endothelial cells protrude extensions into the lumen and form abluminal sprouts, with leading tip cells penetrating deep into the tissue. These endothelial cells are often leaky, have wide junctions, and contain multiple fenestrations and other transendothelial channels, resulting in haemorrhage and increased interstitial fluid pressure, which limits perfusion [8, 37]. The basement membrane shows conspicuous structural abnormalities, including a loose association with endothelial cells and pericytes, broad extensions away from the vessel wall, and multiple layers visible by electron microscopy [38]. Tumor pericytes are also implicated in the abnormal nature of tumor vessels. Pericytes around tumor vessels are loosely attached to endothelial cells, have abnormal shapes, or present long cytoplasmic processes away from the vessel wall. Strategies to enhance pericyte coverage may prevent tumor vessel leakage, dilatation, and tortuosity and may promote vessel stabilization and normalization [7].

Tumor blood flow is not only chaotic, but also stagnant in places and, because of continuous vessel remodeling, it is variable between different tumors, between the primary cancer and its metastatic lesions, and within the same tumor [39]. The tumor microenvironment, because of hypoxia, low pH, and high interstitial fluid pressure, can alter the intrinsic characteristics of tumor cells, resulting in a selection of tumor clones and facilitated escape of neoplastic cells through leaky vessels [37], favoring the metastasis.

## 3. Mechanisms of Angiogenic Activation

Tumor-induced angiogenesis depends on the production of pro-angiogenic growth factors by the tumor cells [6] and involves a diverse array of molecules that includes both those that regulate the maintenance and destruction of the perivascular milieu (which includes both extracellular matrix and perivascular cells) as well as those which stimulate endothelial cell division and migration [12].

The first angiogenic growth factor, basic fibroblast growth factor (bFGF), belongs to the FGF family. bFGF stimulates all major steps in the angiogenesis cascade and is produced by many cells, among which are macrophages and tumor cells. Although FGF does not have a signal sequence that allows regular secretion, it is released in the extracellular matrix after which angiogenesis is initiated. bFGF is a pleiotropic mitogen for growth and differentiation, known to be involved in endothelial cell proliferation, extracellular matrix degradation, endothelial cell migration, and modulation of junctional adhesion molecules [40].

The angiopoietin family, another important growth factor family in angiogenesis, includes three members (in humans), namely angiopoietin-1 (Ang-1), angiopoietin-2 (Ang-2), and angiopoietin-4 (Ang-4) that all bind to the endothelial tyrosine kinase receptor Tie-2. The most remarkable characteristic of this family is the opposing effect of the different ligands binding to the same receptor. Ang-1 activates the Tie-2 signalling while Ang-2 inhibits this activation. Ang-1 is involved in endothelial cell migration, adhesion, and the recruitment of pericytes and smooth muscle cells, while Ang-2 is vessel destabilizer [40]. Ang-1 is produced by numerous cell types including mural cells (pericytes, smooth muscle cells), fibroblasts, and monocytes, thereby acting in a paracrine manner; Ang-2 is almost exclusively produced by endothelial cells [41]. The role of Ang-1 in tumor angiogenesis is controversial. In fact, its overexpression stimulates divergent responses depending on tumor type, ranging from promotion to limitation of growth [42].

Ang-2 is the main angiopoietin ligand in tumors [4]. Its levels are upregulated by hypoxia [43]. Ang-2 overexpression in mammary carcinoma cells induces intratumoral hemorrhage and nonfunctional and abnormal blood vessels [44]. It acts in connection to VEGF, and the functional correlation of the coordinated VEGF/Ang-2 activity is an increase in host vessel permeability, loss of blood-brain-barrier function in cerebral vessels, microvascular dilation, and sprout formation. In the later stage of tumor development, Ang-2 and VEGFR-2 continue to be expressed at high levels by the host and tumor microvasculature, which remain in a state of angiogenic plasticity [13].

The role of inflammation and inflammatory cells in tumor development and progression has been increasingly studied. Circulating leukocytes, red blood cells, and platelets participate in angiogenesis by VEGF secretion [45, 46], and this explains angiogenesis in tumors of blood cells. Besides VEGF, activated platelets also release other angiogenic proteins, including FGF, insulin-like growth factor 1 (IGF-1), and platelet-derived growth factor (PDGF) as well as Ang, stromal cell-derived factor-1 (CXCL12), MMP- (metalloproteinase-) 1, MMP-2, and MMP-9 [47]. VEGF, bFGF, epidermal growth factor (EGF), PDGF, and TGF-*α*, can also be produced by tumor associated macrophages [48]. After recruitment and activation, macrophages secrete a broad spectrum of growth factors, cytokines (such as IL-1, IL-2, IL-6, IL-8, IL-12, and IL-17) [49], chemokines, and matrix-degrading enzymes, which are directly involved in the endothelial cell function and facilitate endothelial cell migration via extracellular matrix remodeling. In a hypoxic environment such as that in tumors, hypoxia tightly regulates the expression of various pro-angiogenic chemokines in macrophages, including CXCL12, C-C chemokine ligand 2 (CCL2), CXCL8, CXCL1, CXCL13, and CCL5 [48].

In the mosaic of cells and substances promoting angiogenesis, an important role is played by cancer stem cells (CSC) [50]. These cells are distinguished from other substances in that they can reconstitute in a recipient animal a tumor that is identical to the parental patient tumor and that can be serially xenotransplanted indefinitely. CSC produce high levels of VEGF in both normal and hypoxic conditions, leading to a strong angiogenic response, which can promote tumor growth [49].

Vascular endothelial cell growth factor (VEGF) or vascular permeability factor is the most studied in the stimulation of angiogenesis. It increases the expression of MMPs and plasminogen activators for the degradation of the extracellular matrix and subsequently endothelial cell migration [51].

**Figure 3:**
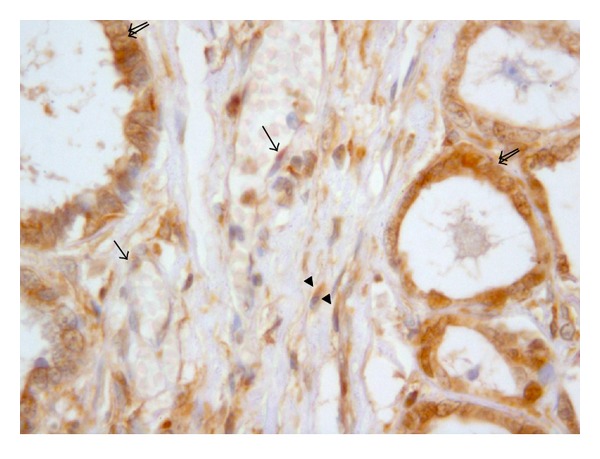
Dog. Mammary tumor. Cytoplasmic endothelial (arrow) positivity of vessel walls, of stromal fibroblasts (arrowhead), and of carcinoma cells (double arrows). Anti-VEGF immunohistochemistry x63.

**Figure 4:**
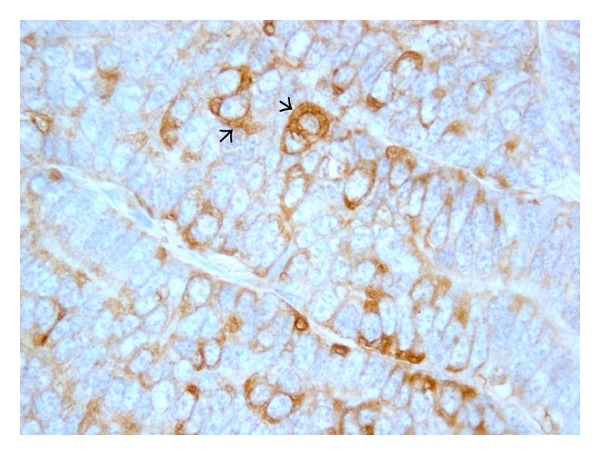
Dog. Mammary tumor. Cytoplasmic positivity (arrow) in carcinoma cells. Anti-VEGF immunohistochemistry x63.

VEGF denotes a family of five related mammalian growth factors: VEGFA (the prototype), VEGFB, VEGFC, VEGFD, and PLGF (placental growth factor) [52]. The role of this latter is underestimated, even if it is known that activated endothelial cells produce large amount of PLGF, thereby regulating the VEGF mediated angiogenic switch [40]. Several of the VEGF family of ligands and receptors, notably VEGFA, are regulated by HIF (hypoxia-inducible factor) [53], linked to the hypoxic condition present in the necrotic compartments [54]. VEGF protein is synthesized and localized in the cytoplasmic granules of neoplastic epithelial cells, endothelial cells, and stromal cells, (Figures 3 and 4) indicating that both autocrine and paracrine signaling induces proliferation of endothelial sprouts [55, 56].

## 4. Overview of Squamous Cell Carcinoma in Humans, Dogs, and Experimental Animals

Squamous cell carcinoma (SCC) is the second most common skin tumor in humans and dogs [57] following basal cell carcinoma and mast cell tumor, respectively. In Sprague-Dawley rats, SCC is the 2nd most common cutaneous epithelial neoplasm [58–60], and in mice SCC has rarely been reported since creosoted wooden cages have been discarded [61]. In humans, cutaneous SCCs commonly arise directly from or in close proximity to actinic keratosis [62]. Similarly, actinic keratoses and SCCs often coexist in dogs [57]. In experimental animals, actinic keratosis and SCC can be induced by chronic UV radiation [63]. Genetic mutations in the p53 tumor suppressor gene have been reported in SCC in humans, dogs, and experimental animals [64–67]. Other mutations have been reported in humans and later reproduced in experimental animal models, but are yet to be found in dogs [67, 68].

## 5. Vascular Endothelial Growth Factor Mediated Angiogenesis in Squamous Cell Carcinoma

Angiogenesis is essential for the growth and metastases of many malignant tumors including cutaneous squamous cell carcinoma. Several studies have shown that VEGF plays an important role in cutaneous malignancies. In human skin VEGF is expressed at low levels within the normal epidermis. The production of VEGF by normal keratinocytes can be induced by many stimuli including the tumor promoter 12-O-tetradecanoylphorbol-13 acetate (TPA), UV radiation, keratinocyte growth factor, hypoxia, and transforming growth factor-*α* [69–72]. The expression of VEGF is markedly increased within epithelial tumors of the skin [73–75]. In particular, cutaneous SCCs display intense and widespread expression, with the highest expression in tumor cells close to inflammatory foci [74]. Furthermore, the expression of VEGF is higher in poorly differentiated SCCs compared to well-differentiated tumors [76].

VEGF is also expressed in the normal canine epidermis [77]. Only two studies examined the expression of VEGF in canine cutaneous SCC. The expression of VEGF was found in the majority of SCCs in both studies [78, 79]. Both studies also reported an increase in VEGF expression level with the advancement of histological grade with one study reporting higher levels in SCC arising on the toe [78].

Several experimental animal models have been used to study SCC. These include mice xenograft models, chemically induced SCC mice models, and genetically engineered mice models [80–82]. In the cutaneous two-stage chemical carcinogenesis mouse model, hyperplastic epidermal lesions develop initially. These lesions progress to benign papillomas first and later become squamous cell carcinomas [83]. The progression of lesions in this model is associated with a sequential increase in VEGF at the mRNA and protein level [83]. Thus, similar to humans and dogs the expression of VEGF in mice is low within the normal epidermis and increases stepwise during carcinogenesis [83]. Moreover, examination of the vasculature using the endothelial cell marker CD31 has shown that vascular density increases considerably in the early papilloma stage [84]. In more advanced papilloma lesions, the vascular density stabilizes but the size of the vasculature is increased [85]. The use of VEGF transgenic mice models overexpressing VEGF in epidermal keratinocytes, such as K6-VEGF and K14-VEGF transgenic mice, has provided strong evidence for the involvement of VEGF in angiogenesis [86, 87]. Compared to controls, these VEGF transgenic mice show marked increase in vascular density in the normal skin and in skin tumors. Furthermore, these transgenic are more susceptible to two-step chemical carcinogenesis [86, 87].

In dogs with cutaneous SCCs, one study reported a significant difference in vascular density among different histological grades with higher grade tumors having higher vascular density [78]. Contrary to this finding, another study failed to find a correlation between VEGF expression level and tumor grade [79]. The disparity between the two studies could be due to the difference in endothelial markers used to assess vascular density. Additional studies, utilizing larger sample size, are needed to examine the relationship between VEGF and vascular density in canine cutaneous SCCs.

## 6. Autocrine VEGF Signaling in SCCs

An autocrine pathway involves the secretion of a ligand which, consequently, engages a specific receptor on the same cell surface and results in a signaling response that affects cell function [88]. It is now clear that tumor cells acquire a certain degree of self-sufficiency through autocrine signaling pathways that facilitate their growth, survival, and invasion [89]. One of the first indications that VEGF may exhibit autocrine function in carcinoma was provided in a study on invasive breast carcinoma cell lines. Lowering VEGF expression by 50% using antisense oligonucleotides resulted in a significant increase in apoptosis, even in the presence of 10% serum [90]. Similar to breast carcinoma, several studies reported an autocrine role for VEGF in cutaneous SCCs. Using the K5-SOS transgenic mouse model, in which mice develop skin tumors spontaneously or after a skin wound, it has been shown that epidermal tumor cells of K5-SOS transgenic mice express high levels of VEGF and its receptors Flt1 and Nrp1 [91]. Deletion of VEGF in this mouse model resulted in reduced tumor development, vascular density, and tumor proliferation [91]. The main source of VEGF in the skin is epidermal keratinocytes [92], although other cell types including macrophages and fibroblasts are known to produce VEGF [93, 94]. Several VEGF receptors have been identified on the surface of epidermal keratinocytes including VEGFR-1, VEGFR-2, and NRP-1 [95]. VEGFR-1 has also been localized to human and mouse skin tumors and in SCC cell lines [91]. VEGFR-1 expression was detected in K5-SOS mice and deletion of VEGFR-1 in these mice resulted in decreased development of papilloma and decreased tumor cell proliferation. Furthermore, SCC cell lines in which VEGFR-1 was deleted exhibited lower cell proliferation [91]. These studies point to an autocrine role of VEGF in skin carcinogenesis in which VEGF results in enhanced tumor cell proliferation by binding to VEGFR-1.

In dogs VEGFR-2 was detected in a few canine tumors including cutaneous SCC, simple mammary gland adenocarcinoma, fibrosarcoma [59, 60, 79], apocrine gland anal sac adenocarcinoma, and thyroid carcinoma [96]. Interestingly, the expression of VEGF and VEGFR-2 in canine cutaneous SCC was positively correlated with tumor cell proliferation index [79]. This indicates that VEGF may enhance tumor cell proliferation through an autocrine loop which involves VEGFR-2. Additional *in vitro *and* in vivo* studies are needed to examine the autocrine role of VEGF and the roles of its receptors in canine cutaneous SCC.

## 7. Angiogenesis in Canine Mammary Tumors

Mammary tumor growth forces cells to face the same increasing demand for oxygen, glucose, amino acids, and waste exchange as do normal mammary cells during development, different oestrus phases, and lactation [97]. As in normalcy, such a demand drives angiogenesis, the process whereby new blood vessels sprout from the existing vasculature [98]. Several molecules such as cell surface receptors, growth factors, and enzymes are involved in this process.

Tumor-associated angiogenesis and lymphangiogenesis, the process whereby lymphatic vessels are generated, are crucial in mammary tumor progression. Tumor vasculature, together with the lymphatic system, is the main route through which BMDCs (bone marrow-derived dendritic cells) infiltrate the tumors and tumor cells themselves are able to evade from primary sites and systemically disseminate [99].

The VEGF and its receptors VEGFR1 and VEGFR2 are leading players in angiogenesis [100]. The literature is somewhat conflicting in what regards VEGF expression in CMT (canine mammary tumors) and its relationship to the biological behavior of this type of tumors. This is most likely due to the antibodies used which recognize distinct splicing forms of the protein [54, 56, 101, 102]. Other possible explanation for the inconsistency of the results lays on the fact that VEGF-A has alternative splicing forms some of which are more prevalent than others and correlate to worse prognosis in different types of cancer. These distinct isoforms are known to induce distinct vessel conformation and type of circulation. The receptor tyrosine kinase (RTK) activity of VEGFR1 is also dependent on the isoform present [103]. Thus, given that distinct splicing forms differ in their function, this may account for the differences in findings from different groups studying VEGF in CMT. Regardless of this, all authors find a high expression of VEGF in CMT cells [54, 56, 101, 102]. Interestingly, work in CMT showed an upregulation of VEGF, assessed by its level of intensity, at the invasive front of the primary tumor cells and also in cells surrounding necrotic areas. The latter pointing to a potential role for the harsh microenvironment found in these areas in regulating VEGF [56]. These are hypoxic regions of the tumors in which the cells suffer from lack of oxygen but also glucose and amino acids deprivation, being that the lactate concentration is high at all times. Low levels of oxygen trigger stabilization of the hypoxia-inducible factor (HIF) *α* subunits. In the absence of oxygen, the *α* subunits are not hydroxylated and hence are no longer targeted for degradation by the proteosome. Stabilized HIF-1 *α* upregulates several genes in order to promote survival under hypoxic conditions. Among these is the VEGF. Remarkably, in a subsequent work, de Oliveira et al. [104] have observed that the epidermal growth factor receptor (EGFR) is significantly associated with the presence of necrosis in CMT and was found to be overexpressed in these same stress-inducing areas. EGFR has a well-established role in mammary angiogenesis and was found to be overexpressed in tumors which relapse to antiangiogenic therapy [105, 106].

Other HIF-1 *α* target genes interestingly expressed in CMT are galectin-1 and galectin-3. Galectin-1 and galectin-3 are members of galectins, a carbohydrate-binding family of proteins. These are involved in cell survival to anoikis, cell-cell, and cell-ECM (extra-cellular matrix) adhesion and are also crucial players in angiogenesis. Galectin-3 is known to be chemoattractant to endothelial cells and to stimulate neovascularization *in vivo*, therefore contributing to tumor angiogenesis, an essential step for metastatic spreading [107]. Galectin-3 is in accordance overexpressed in necrosis-surrounding cells both in primary and in metastatic CMT lesions despite downregulated in other tumor areas. It is of note that galectin-3 was also found to induce VEGF in human breast cancer cells and hence promote angiogenesis [108]. Tumor cells secrete galectin-1 in order to induce angiogenesis [109]. This galectin is overexpressed both in tumor cells and stroma of CMT [110]. Galectin-1 was interestingly recently found to be the target of the potent angiogenesis inhibitor Anginex [111].

ECM is deposited to form a basement membrane to surround the blood vessels during tumor angiogenesis. Importantly, however, the basement membrane of the tumor vasculature is more porous and leaky than normal [112, 113], which facilitates tumor cell metastasis and immune cell infiltration and promotes cancer progression [114, 115]. The ECM plays a crucial role in blood vessel formation. In addition to guiding endothelial cell migration and branching, ECM and its fragments may be involved in endothelial cell survival and proliferation to supply cellular building blocks for vessel growth [116]. ECM biomechanical properties appear to play an especially important role in angiogenesis. Indeed, vascular networks with markedly distinct branching patterns have been observed when endothelial cells are grown on matrix with different elasticity [117]. Among other changes, ECM biomechanical properties may be influenced by posttranslational modifications on ECM proteins such as altered glycosylation, a common feature in cancer. Malignant CMT displayed an altered ECM glycosylation which correlated with the downregulation of GLT25D1, a *β* (1-O) galactosyltransferase that modifies collagen [110]. In parallel with the downregulation of galectin-3, malignant CMT displayed an overall loss of galectin-3-binding sites in the ECM and focal expression of galectin-3-binding sites mainly detected in intravascular tumor cells and endothelium. Interestingly, GLT25D1 mRNA expression was strikingly downregulated in malignant CMT-U27 compared with the benign cell line, and its expression was further decreased in a galectin-3 knockdown CMT-U27 cell line. ECM components are involved in cellular morphogenesis, including vessel lumen formation [118] and other aspects of tubulogenesis during tumor angiogenesis [98]. Moreover, ECM fragments, derived from collagens types IV and XVIII, have potent stimulatory or inhibitory effects on angiogenesis. These are likely to collaborate with other pro- or antiangiogenic factors, including VEGF, to determine the architecture of vessel-branching [119]. It is of note that, to initiate vascular branching, vessel basement membrane ECM needs to be fragmented and removed. This process is most likely performed by MMPs which are expressed by invading endothelial cells or can be secreted by the tumor cells. MMPs are also required for the top edge of an endothelial branch, to overcome and progress throughout the stromal tissue towards distressed cells [29, 120]. MMP2 and MMP9 are two members of this family well known to contribute to angiogenesis. Benign CMT present MMP-2 immunoreactivity in the myoepithelial cells lining the basement membrane of tubuloalveolar structures, while malignant CMT showed mainly diffuse expression in neoplastic cells [121]. Stromal-associated MMP9 which mediates tumor-induced angiogenesis showed higher expression in highly proliferative CMT and in CMT with invasive growth, high histologic grade, and metastatic capacity [122].

The serine protease urokinase-type plasminogen activator (uPA) involved in the control of extracellular matrix turnover is involved in vascular remodeling and was also shown to be implicated in the stimulation of angiogenesis [123]. Malignant CMT expressed significantly more uPA than benign tumors. In malignant CMT, high uPA stromal expression was significantly associated with larger tumor size, high Ki-67 expression, invasive growth, high histological grade, regional lymph node metastases, development of distant metastases, and lower overall survival (OS) and disease-free survival (DFS) [121].

Antiangiogenic therapy for breast cancer has raised a great deal of controversy within the scientific community over the last years. For instance, bevacizumab blocks VEGF, its association with paclitaxel doubles the time of human breast cancer progression although not affecting the patients' overall survival. Therefore there is resistance to antiangiogenic therapy. It is now understood that this type of therapy induces hypoxia and that hypoxia-dependent pathways lead to decreased cell death [124]. The antiangiogenic therapy leads for a while to decreased tumor growth but afterwards relapse occurs. This type of relapse is thought to be dependent on two distinct mechanisms (1) altered cell behavior with increased invasion and metastatic capacity and (2) reneovascularization. The first mechanism can be explained by the fact that by inhibiting VEGF hypoxia is induced but there is also an increase in Met phosphorylation which is usually inhibited by VEGF. Increased Met signaling leads to an upregulation of epithelial mesenchimal transition (EMT) related genes such as Snail, N-cadherin, vimentin, and CD44. The second mechanism relates to a hypoxia-induced increase in tumor-infiltrating bone marrow derived cells, vascular progenitor cells able to differentiate into endothelial cells, implicated in antiangiogenic treatment resistance [125, 126].

In conclusion, there are several interdependent pathways driving angiogenesis in cancer in general and in CMT in particular. This knowledge is of the utmost importance when considering future antiangiogenic therapies for CMT management in veterinary oncology.

## 8. Angiogenesis Models for Preclinical Drug Studies

Preclinical drug studies theoretically should define whether a particular potential therapy has activity against tumors with the appropriate drug target, and whether or not activity observed in these models can be extrapolated to humans. The interpretation of data from preclinical studies is often perceived as a bottleneck in drug development; thus, the selection of appropriate preclinical angiogenesis models with reproducible activity can lead to subsequent success in the clinic.

The emergence of targeted therapy has resulted in the need for translational preclinical drug assessment strategies in an attempt to bridge the gap between preclinical models and clinical efficacy. Existing models that have been used in the development of traditional cytotoxic drugs should be reevaluated and refined for these newer “molecularly targeted drugs.” It remains critical to choose appropriate angiogenesis assays to evaluate the efficacy of novel drug compounds and to identify potential targets within the angiogenic disease to enable the proper translation of data from preclinical to the clinic testing. The principle angiogenesis assays include those for endothelial cell proliferation, differentiation, migration, and coculture models *in vitro*; vessel outgrowth from organ cultures such as the *ex vivo* rat aortic ring assay; and *in vivo* assays such as chick chorioallantoic membrane (CAM), zebrafish, sponge implantation, corneal, dorsal air sac, chamber, and tumor angiogenesis models (reviewed in [127]).

With respect to animal models, two of the most commonly used for preclinical testing are tumor xenograft models (cell line xenograft or patient-derived xenograft), which involves transplantation of human or syngenic mouse tumor cells either subcutaneously or at a relevant orthotopic site in immunocompromised mice, and genetically manipulated mice that develop spontaneous lesions (genetically engineered mouse models (GEMMs)), which involves genetic engineering to introduce genomic alterations that drive cancers of interest, ideally confined to the relevant tissue. When choosing a mouse model for preclinical testing, consideration is given to the tumor microenvironment targeted by the drug, whether the drug is intended for early-stage versus late-stage therapy and whether modeling the metastatic spread is necessary. GEMMs have been especially effective for studying the early events in tumorigenesis; however, they have not replaced xenograft models as reliable clinically predictive tools for examining the efficacy of therapeutic approaches to treat metastatic disease. After the *Rip1*-*Tag2 *tumor model was characterized as highly angiogenic [19], this and other GEMMs were used extensively to evaluate antiangiogenic therapies *in vivo*. While many of these models show single-agent activity, predicting the clinical activity and efficacy of antiangiogenics from animal data has proven challenging since the translation of the single-agent activity has not been seen in clinical trials targeting advanced metastatic disease with the exception of renal cell, hepatocellular, and ovarian carcinoma [128]. For example, TNP-470, a synthetic analog of the antiangiogenic agent fumagillin, exhibited significant tumor regression in the *Rip1*-*Tag2 *model as a single agent [129]; however, its efficacy in humans was very limited, due to its short half-life and neurotoxic effects [130]. Early preclinical studies have shown that monoclonal antibodies (mAbs) targeting VEGF-A suppress growth of several human tumor xenograft models [131]. Other antiangiogenic agents including mAbs against VEGFR2, soluble VEGF receptors, and small-molecule inhibitors of the VEGF receptor tyrosine kinases (RTKs) were also found to inhibit tumor growth in xenografts and GEMMs [128]. While these inhibitors demonstrated strong single-agent activity when the treatment was initiated at early stages of tumorigenesis, most clinical trials have been conducted in patients with advanced disease. These VEGF pathway inhibitors have generally proven clinically efficacious, not broadly as single agents, but rather in combinations with chemotherapy that is cancer type-specific.

Indeed experience with experimental therapeutics in the *Rip1*-*Tag2* model over time suggests that rational trial design combined with more specific drug targets can improve the predictive power of preclinical studies. As advances in cancer genomics lead to more pharmacogenetics testing, preclinical model systems need to continually be refined to define and validate biomarkers to aid in patient selection and identify antiangiogenic predictive markers of response and resistance. Careful extrapolation of preclinical data in an era of increasingly specific agents, more sophisticated animal models, and increased understanding of the molecular drivers of cancer is required. Successful translation of preclinical data to the clinic has been demonstrated with the approval of several antiangiogenic agents over the last decade.

## 9. Clinically Approved Antiangiogenic Therapies

The angiogenic pathway has been a valid target for anticancer drug development and VEGF has become the primary antiangiogenic drug target. Investigation of hundreds of potential angiogenesis inhibitors resulted in the approval of bevacizumab (VEGF neutralizing antibody) by the U.S. Food and Drug Administration as the first antiangiogenic agent. Bevacizumab is approved in combination with chemotherapy or cytokine therapy for advanced metastatic cancers including colorectal cancer (CRC), nonsquamous non-small-cell lung cancer (NSCLC), and renal cell cancer (RCC). Bevacizumab monotherapy is approved for recurrent glioblastoma. Subsequent antiangiogenic agent FDA approvals include the multitargeted pan-VEGF receptor (VEGFR) tyrosine kinase inhibitors (TKIs) that target different parts of the angiogenic pathway: sorafenib for the treatment of advanced RCC and unresectable hepatocellular carcinoma; sunitinib for metastatic RCC, gastrointestinal stromal tumor, and advanced pancreatic neuroendocrine tumors; pazopanib for metastatic RCC and advanced soft tissue sarcoma (with orphan drug status designation for this indication); vandetanib for advanced medullary thyroid cancer; and axitinib for advanced RCC. More recently, ziv-aflibercept was approved for use in combination with chemotherapy for metastatic CRC. Ziv-aflibercept, a protein comprised of segments of the extracellular domains of human VEGFR1 and VEGFR2 fused to the constant region (Fc) of human immunoglobulin G (IgG1), functions as a soluble decoy receptor that neutralizes VEGFs.

In general, these VEGF/VEGFR inhibitors provide modest clinical benefit in terms of prolonging progression-free survival (PFS) or overall survival (OS) of cancer patients with a median duration of response in weeks to months. Moreover, clinical experience with bevacizumab has proven to be rather perplexing with improvement in PFS but little to no benefit in OS. Of the 16 pivotal phase III trials conducted with bevacizumab in solid tumors only three were positive for OS first-line mCRC [132], second-line mCRC [133], and first-line recurrent/advanced NSCLC [134]. This concern made headlines in 2011 when the FDA rescinded the approval of bevacizumab for the treatment of breast cancer following the failure of two phase III clinical trials to demonstrate an improvement in OS for first-line breast carcinoma [135, 136].

## 10. Combination Therapies

Tumor angiogenesis is a highly complex process involving multiple growth factors and their receptor signaling pathways as well as key cellular players in the microenvironment such as pericytes, endothelial cells, and bone marrow-derived precursors. Based on current evidence, effective therapy will probably rely on a combination approach that involves simultaneous targeting of multiple angiogenic pathways coupled with cellular targets in the microenvironment. Whether other targeted agents exhibit beneficial effects when combined with VEGF inhibitors remains to be investigated; however, the use of targeted agents combined with conventional therapy is intended to increase tumor response without significant increases in toxicity.

A number of studies have shown that antiangiogenic agents in combination with chemotherapy or radiotherapy result in additive or synergistic effects. Several models have been proposed to explain the mechanisms responsible for the chemosensitizing effects of antiangiogenic therapy [137]. One hypothesis is that antiangiogenic therapy may normalize the tumor vasculature, thus resulting in improved oxygenation, better blood perfusion, and, consequently, improved delivery of chemotherapeutic drugs [37]. A second model suggests that chemotherapy delivered at low doses and more frequent intervals with no extended drug-free break periods (also called metronomic chemotherapy) preferentially damages endothelial cells in the tumor neovasculature [138, 139], suppresses circulating endothelial progenitor cells [140, 141], sustains antiangiogenic activity, and reduces acute toxicity [142]. Finally, the third model addresses the use of antiangiogenic drugs to slow down tumor cell repopulation between successive cycles of cytotoxic chemotherapy [143]. This model underscores the importance of timing and sequence in achieving the maximal therapeutic benefit from combination therapies. Nonetheless, it remains a challenge to determine why bevacizumab has proven largely ineffective as a single agent whereas VEGFR TKIs have repeatedly failed in randomized phase III trials when used in combination with chemotherapy. Additionally, vascular disrupting agents which aimed at collapsing the existing vascular structures in combination with traditional antiangiogenic agents may further improve the overall antitumor effect [144, 145]. Ongoing studies will need to evaluate the most effective combination of antiangiogenic agents with other targeted therapies and/or conventional therapies in order to improve clinical outcomes.

## 11. Challenges to Antiangiogenic Therapy

### 11.1. Surrogate Markers of Tumor Angiogenesis and Antiangiogenic Therapy

Antiangiogenic therapy has fostered the quest for surrogate markers of response or resistance to assess and monitor the clinical effects of these inhibitors. The lack of validated biomarkers to date remains a challenge, thereby limiting the successful use of antiangiogenic therapy in the clinic. Surrogate markers are important for guiding the clinical development of these agents and to select patients most likely to benefit from this therapeutic approach. A number of candidate biomarkers including genetic, tissue, imaging, and circulating biomarkers are emerging that need to be prospectively validated [146, 147]. Several mechanisms are currently being investigated and include tumor biopsy analysis, microvessel density, noninvasive vascular imaging modalities (positron emission tomography, dynamic contrast-enhanced MRI), and measuring circulating biomarkers (levels of angiogenic factors in serum, plasma, or urine; circulating endothelial cells and their precursors) [148].

Recent research efforts have focused on identifying genetic and toxicity biomarkers to predict which patients will benefit from anti-VEGF/VEGFR therapy and identify patients at risk of adverse events. The existence of VEGF single nucleotide polymorphisms (SNPs) and their association with clinical outcome may be predictive markers of response to bevacizumab. In a metastatic breast cancer study involving patients being treated with paclitaxel and bevacizumab (E2100 trial), SNP analysis demonstrated that the VEGF-2578 AA and VEGF-1154 AA genotypes predicted an improved median overall survival, whereas the VEGF-634 CC and VEGF-1498 TT variants predicted protection from grades 3-4 hypertension in the combination treatment arm [149]. Another potential candidate surrogate marker is hypertension, one of the most common toxicities in patients taking VEGF inhibitors. The degree of hypertension can serve as a biomarker of survival and show predictive value for antitumor efficacy in patients after bevacizumab or TKI treatment. In the same E2100 trial, patients who experienced grade 3 or 4 hypertension survived significantly longer, although hypertension was seen in patients with the VEGF-634 CC and VEGF-1498 TT genotypes [149].

Additional biomarkers of response to antiangiogenic therapy include measuring circulating elevated VEGF and placental growth factor levels [146], while biomarkers of resistance include circulating basic fibroblast growth factor, stromal cell-derived factor 1*α*, and viable CECs increased when tumors escaped treatment [150]. If validated, these findings could help select for subgroup of patients who may benefit from antiangiogenic therapy and lead the way to possible future personalizing of antiangiogenic therapy.

### 11.2. Resistance to Antiangiogenic Therapy

The clinical efficacy of angiogenesis inhibitors has recently been met with numerous phase III failures that showed modest survival benefits because tumors elicit evasive resistance [151]. Understanding the resistance mechanisms of antiangiogenic therapy is essential to overcome the limited effectiveness of VEGF-pathway inhibitors. Resistance to VEGF pathway-inhibitors may be observed in late stage tumors when tumors regrow during treatment, after an initial period of growth suppression from these antiangiogenic agents. This resistance involves reactivation of tumor angiogenesis and increased expression of other pro-angiogenic factors. As the disease progresses, it is possible that redundant pathways might be implicated, with VEGF being replaced by other (pro)angiogenic pathways, warranting the addition of a second angiogenesis inhibitor that would target these secondary growth factors and/or their activated receptor pathways or the use of a multitargeted pan-VEGFR TKIs. However, resistance to these drugs eventually occurs implicating the existence of additional pathways mediating resistance to antiangiogenic therapies. Whether the administration of angiogenic drugs at earlier stages of the disease may be a more effective and beneficial approach remains to be determined. Moreover, tumor cells bearing genetic alterations of the p53 gene may display a lower apoptosis rate under hypoxic conditions, thereby reducing their reliance on vascular supply and responsiveness to antiangiogenic therapy [152]. The selection and overgrowth of tumor-variant cells that are hypoxia resistant, and thus less dependent [152] on angiogenesis and vasculature remodeling resulting in vessel stabilization [153], could also explain the resistance to antiangiogenic drugs. Other possible mechanisms for acquired resistance to antiangiogenic drugs include tumor vessels becoming less sensitive to antiangiogenic agents, tumor regrowth via rebound revascularization, and vessel cooption [125, 154–158]. Perhaps one of the most intriguing finding is that although endothelial cells are presumed to be genetically stable, they may under some circumstances harbor genetic abnormalities and thus acquire resistance as well [159, 160].

Recent studies report that antiangiogenic therapies induce primary tumor shrinkage and inhibit tumor progression but can also initiate mechanisms that promote tumor invasiveness and metastasis [158, 161, 162]. These mechanisms of resistance to antiangiogenic therapy involve tumor and host-mediated pathways, allowing for differential efficacy in different stages of disease progression [158]. Specifically, antiangiogenic drug resistance mechanisms involve pathways mediated by the tumor, whether intrinsic or acquired in response to therapy or by the host, which is either responding directly to therapy or indirectly to tumoral cues. Angiogenesis inhibitors often cause tumor vessels to regress resulting in vessel normalization. This vascular pruning can also cause intratumoral hypoxia, which in turn activates the hypoxia-inducible 1 factor alpha (HIF-1alpha) and HIF-mediated pathways to promote invasion and metastasis from the tumor [163]. Moreover, several reports have implicated a critical role for hypoxia and HIF in cancer stem cell (CSC) proliferation, self-renewal, and maintenance [164]. The tumor-initiating properties and metastatic potential of CSCs make them key drivers of tumor growth and therapy resistance. Indeed in the recent study, Conley and colleagues demonstrated that hypoxia induced by the administration of angiogenesis inhibitors might accelerate tumor growth and metastasis by increasing the CSC population [165]. This CSC niche may be responsible for mediating tumor metastasis and resistance to cancer treatments. Taken together, antiangiogenic therapy can enhance tumor invasiveness and metastasis to facilitate and/or accelerate disease in microscopic tumors, hence resulting in minimal overall survival advantage. Understanding the mechanisms of resistance, whether intrinsic or acquired, is essential for developing strategies that will allow for optimal exploitation of the potential of angiogenesis inhibitors. It is equally important to identify surrogate markers of resistance to monitor the development of evasive resistance to angiogenesis inhibitors, thereby rendering this therapy more effective in the future.
